# Steerable-wire technique using high-flow steerable microcatheter and 0.025-inch guidewire

**DOI:** 10.1016/j.radcr.2023.08.109

**Published:** 2023-09-22

**Authors:** Mitsunari Maruyama, Hisatoshi Araki, Rika Yoshida, Shinji Ando, Megumi Nakamura, Takeshi Yoshizako, Yasushi Kaji

**Affiliations:** Department of Radiology, Shimane University Faculty of Medicine, P.O. Box 00693-8501, 89-1 Enya cho, Izumo shi, Japan

**Keywords:** Steerable-wire technique, High-flow steerable microcatheter, 0.025-inch guidewire

## Abstract

A high-flow steerable microcatheter has been reported to be useful as a triaxial system. Moreover, the benefits of steerable microcatheters in acute-angle bifurcation vessel insertions and a compact coil-packing technique using intentional folding with a bendable catheter tip have been reported. However, research on the usefulness of a high-flow steerable catheter and 0.025-inch guidewire combination (steerable-wire) technique is lacking. Herein, we report a case of balloon-occluded retrograde transvenous obliteration (BRTO) via the femoral venous approach to illustrate the usefulness of the steerable-wire technique. The steerable-wire technique facilitates the selection of the wire into the target vessel. The steerable-wire can be used instead of the 0.035-inch guidewire, which is versatile as other devices can follow the steerable wire.

## Introduction

High-flow steerable microcatheters (2.9-Fr distal, 2.9-Fr proximal external diameter; Swift NINJA, SB KAWASUMI, Tokyo, Japan) with a steerable tip have been introduced in the market. Once the steerable tip is oriented, the dial stopper can be used to lock the steering dial to maintain the intended direction. Smaller microcatheters (1.7- or 1.9-Fr distal external diameter) are usually used for insertions; a high-flow steerable microcatheter has been reported to be useful as a triaxial system [1], [2], [3]. The benefits of steerable microcatheters in acute-angle bifurcation vessel insertions and a compact coil-packing technique using intentional folding with a bendable catheter tip have also been reported [4], [5], [6]. However, studies on the usefulness of a high-flow steerable catheter and 0.025-inch guidewire combination (steerable-wire) technique are lacking. The steerable-wire technique facilitates the selection of the guidewire into the target vessel. The steerable-wire can be used instead of a 0.035-inch guidewire, which is versatile as other devices can follow the steerable-wire. Herein, we report a case of balloon-occluded retrograde transvenous obliteration (BRTO) via the femoral venous approach to illustrate the usefulness of the steerable-wire technique.

## Case report

### BRTO procedure

**Fig. 1:**
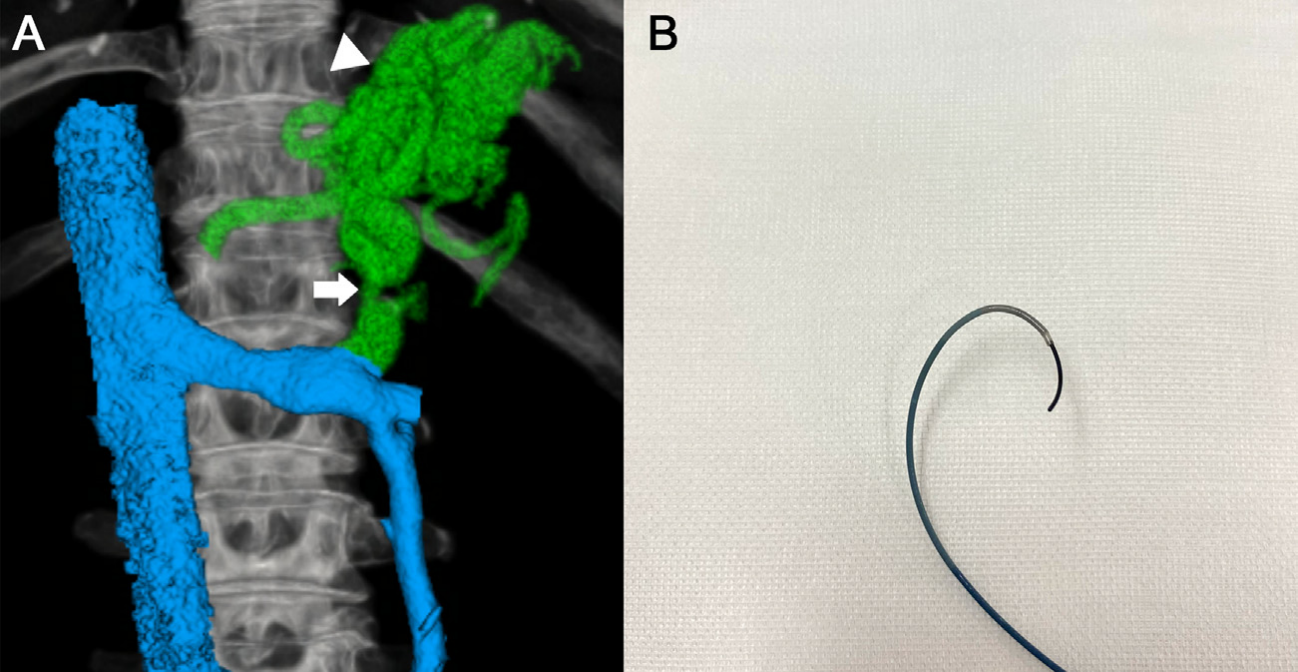
3D VR image reconstructed from CE-CT before BRTO, and steerable-wire. (A) A GR shunt (arrow) and gastric varices (arrowhead) were observed. (B) Both a high-flow steerable catheter and a 0.025-inch guidewire were used as a steerable-wire. 3D VR, 3-dimensional volume rendered; CE-CT, contrast-enhanced computed tomography; BRTO, balloon-occluded retrograde transvenous obliteration; steerable-wire, a high-flow steerable microcatheter and a 0.025-inch guidewire combination; GR shunt, gastro-renal shunt.

A 70-year-old woman with liver cirrhosis associated with hepatitis C virus infection presented to our department of radiology. She had a Child–Pugh score of 6 and a Child–Pugh classification of A. Initial contrast-enhanced computed tomography showed the location of gastric varices with gastrorenal (GR) shunt that could be effectively treated with BRTO (Fig. 1A). We planned to perform BRTO on the patient.

**Fig. 2:**
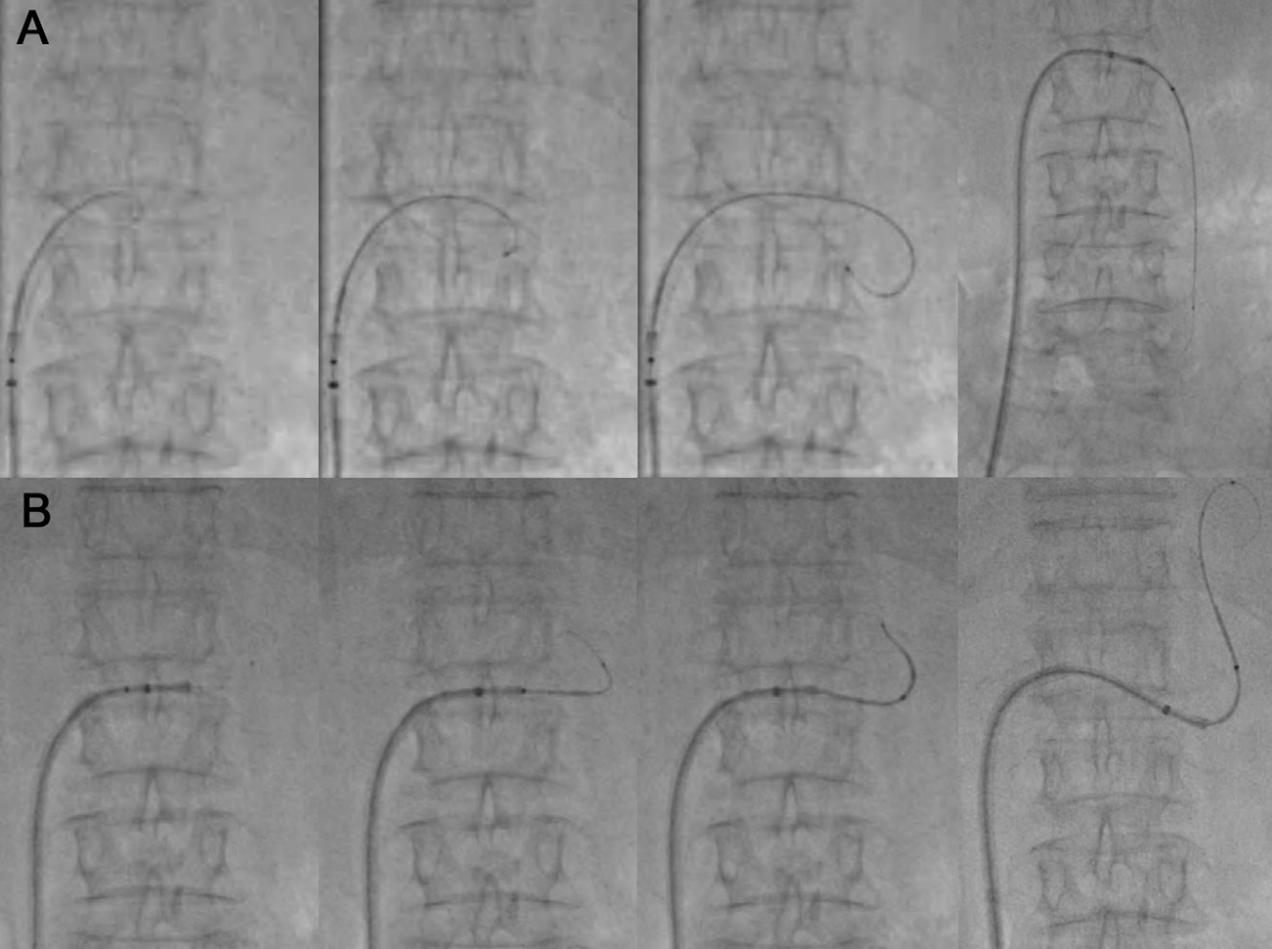
Steerable-wire technique: left renal vein and GR shunt selection. (A) The left renal vein was selected with the steerable-wire at the knuckle locking the steering dial to maintain the intended direction. Then, the left ovarian vein was selected with the steerable-wire, followed by a sheath and a coaxial and double interruption system. (B) A GR shunt was selected with the steerable-wire. The steerable-wire was also used instead of the 0.035-inch guidewire to follow the coaxial and double interruption system. Steerable-wire, a high-flow steerable microcatheter and a 0.025-inch guidewire combination; GR shunt, gastrorenal shunt.

**Fig. 3:**
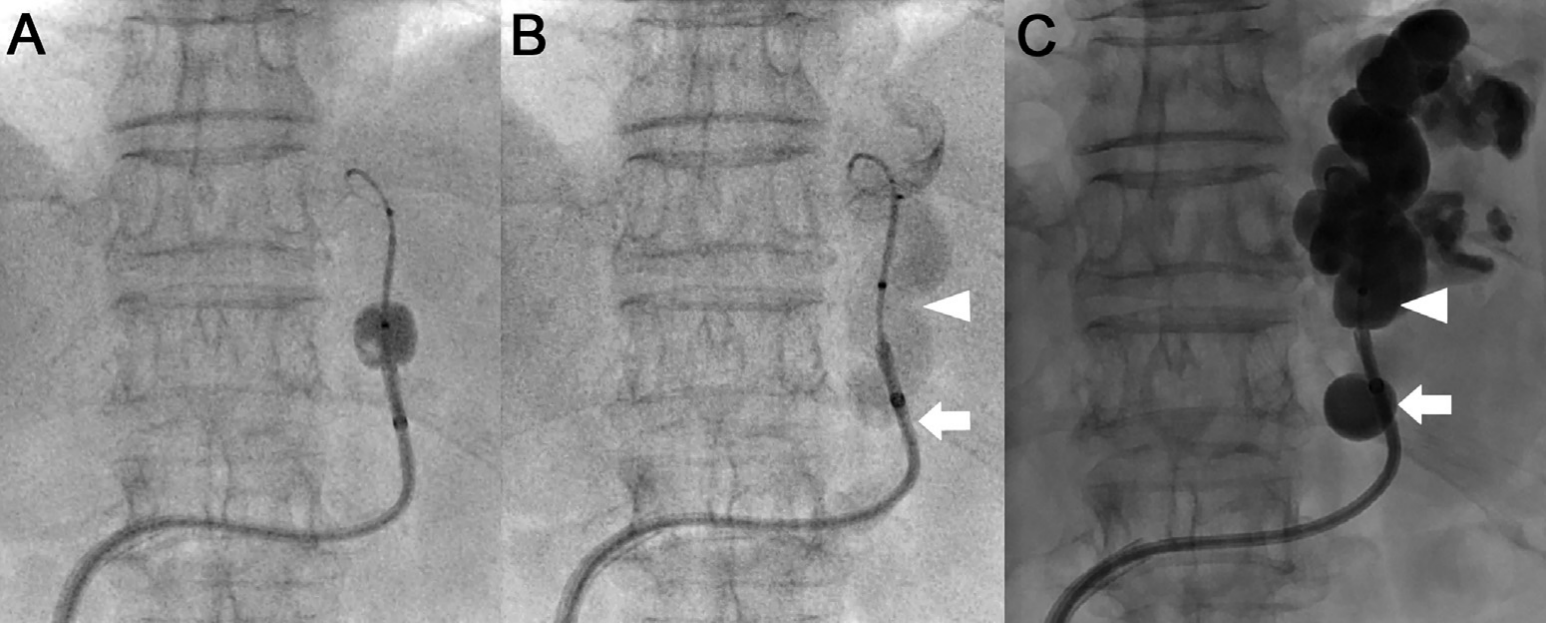
BRTO. (A) The 9-Fr-guiding balloon catheter was followed with the 5-Fr inflated balloon catheter. (B) The 5-Fr-guiding balloon position was adjusted to be over the GR shunt constriction (arrowhead). The 9-Fr-guiding balloon position was adjusted to be over the other GR shunt constriction (arrow). (C) Both balloons (arrowhead, the 5-Fr-guiding balloon; arrow, the 9-Fr-guiding balloon) were inflated after both balloons were fixed at the optimal positions, and BRTO was performed using a sclerosing agent (5% EOI). BRTO, balloon-occluded retrograde transvenous obliteration; GR shunt, gastro-renal shunt; EOI, ethanolamine oleate iopamidol.

Vascular access was achieved percutaneously by establishing an antegrade right common femoral vein puncture. A J-shaped 10-Fr sheath introducer (Super Sheath, Medikit, Tokyo, Japan) was placed at the right common femoral vein. Both a high-flow steerable microcatheter (2.9-Fr distal–proximal external diameter; total effective length of shaft, 150 cm; Swift NINJA, SB KAWASUMI, Tokyo, Japan) and a 0.025-inch guidewire (Radiofocus, Terumo, Tokyo, Japan) were used as a steerable-wire (Fig. 1B). The left renal vein was selected with the steerable wire at the knuckle locking the steering dial to maintain the intended direction. Then, the left ovarian vein was selected with the above steerable-wire system, followed by a sheath and a coaxial and double interruption system (CANDIS; Medikit, Tokyo, Japan) (Fig. 2A). A GR shunt was selected with the steerable-wire; the steerable-wire was also used instead of the 0.035-inch guidewire to follow the CANDIS (Fig. 2B). CANDIS is equipped with a 9-Fr-guiding balloon catheter (balloon diameter: φ20 mm) and a 5-Fr balloon catheter (balloon diameter: φ10 mm), allowing the catheter to be inserted deep into the GR shunt [7]. The 9-Fr-guiding balloon catheter was followed with an inflated 5-Fr balloon catheter (Fig. 3A). The gastric varices were classified as Hirota grade 1 varices by balloon-occluded retrograde transvenous venography [8]. The 5-Fr balloon catheter was inserted deeply into the shunt vessel to find a stable position during the balloon inflation, but a more stable position was determined to be at the constriction of the proximal GR shunt (Fig. 3B, arrowhead). The 5-Fr balloon catheter position was slightly unstable when only inflating the 5-Fr balloon catheter. The 9-Fr-guiding balloon catheter position was adjusted to be over the other constriction of the GR shunt (Fig. 3B, arrow), and the entire system was stabilized by inflating the 9-Fr guiding balloon catheter. We inflated both balloons after the 9-Fr guiding catheter was fixed at the optimal position and performed BRTO using a sclerosing agent (16 mL; 5% ethanolamine oleate iopamidol; Fig. 3C). Overnight balloon inflation was performed. The entire system was removed the next day after the GR shunt embolization was confirmed. No coil placement was performed. A follow-up endoscopy confirmed the disappearance of gastric varices.

## Discussion

We presented a BRTO case to demonstrate the usefulness of the steerable-wire technique via the femoral venous approach. Even if a 0.035-inch guidewire is in the target branch, the pushing maneuver will push the guidewire out, and 4-Fr or larger catheters may not be able to follow the guidewire. Therefore, we have to deeply insert a 0.035-inch wire, which is tricky as the guidewire can push against the varicose veins and cause them to rupture. At the vessel bends, such as the left renal vein-GR shunt, the mechanical support provided by the high-flow steerable microcatheter facilitates deep insertion of the 0.025-inch guidewire. A 0.025-inch guidewire can better reduce varicose vein injuries compared to the 0.035-inch guidewire. Moreover, the high-flow steerable microcatheter can easily follow a 0.025-inch guidewire and, as an alternative to a 0.035-inch guidewire, a 4-Fr or larger catheter device can easily follow a steerable-wire. Using a high-flow steerable microcatheter as a triaxial system allows the coil to be placed deeper into the target vessel. Furthermore, a single high-flow steerable microcatheter is not as stiff as a 0.035-inch guidewire, but a 0.025-inch guidewire can be used to achieve stiffness comparable to that of a 0.035-inch guidewire.

The advantage of CANDIS is that the 9-Fr guiding balloon catheter acts as a guide to improve the operability of the 5-Fr balloon catheter. The 9-Fr guiding balloon catheter can be followed with the 5-Fr inflated balloon catheter, enabling the insertion of the catheter deep into the GR shunt. The Hirota classification grade can then be improved [8]. When complete balloon occlusion by a 5-Fr balloon catheter could not be achieved, complete balloon occlusion by a 9-Fr guiding balloon catheter is possible. In the present case, the 5-Fr balloon catheter position was slightly unstable; thus, the 9-Fr guiding balloon catheter was also inflated in case the 5-Fr balloon catheter was dislodged. Therefore, it can also be used as a secondary system to prevent serious complications, such as pulmonary embolism.

A high-flow steerable microcatheter has technical limitations. First, the total effective length of the catheter shaft is limited to 150 cm, and no other lengths have been introduced in the market. Therefore, the catheter may not reach vascular branches that are far from the puncture site. Second, the use of a steerable catheter takes some getting used to. However, once you get used to it, the torque is more effective than a small-diameter steerable microcatheter (2.0 or 2.4-Fr external diameter); thus, it is useful. The strengths of the steerable-wire are high versatility in target vessel selection, but the weakness is that the equipment cost is higher than that of a single 0.025-inch wire.

## Conclusion

The high-flow steerable microcatheter and 0.025-inch guidewire combination (steerable-wire) technique facilitates the selection of the wire into the target vessel. The steerable-wire can be used instead of the 0.035-inch guidewire, which is versatile as other devices can follow the steerable-wire.

## Author contributions

All authors provided substantial contributions to the manuscript and approved the final version of the article to be published.

## Patient consent

Informed consent was obtained for the publication of this case report.
